# 3D genome-selected microRNAs to improve Alzheimer's disease prediction

**DOI:** 10.3389/fneur.2023.1059492

**Published:** 2023-02-13

**Authors:** Keyi Li, Runqiu Chi, Liangjie Liu, Mofan Feng, Kai Su, Xia Li, Guang He, Yi Shi

**Affiliations:** ^1^Key Laboratory for the Genetics of Developmental and Neuropsychiatric Disorders, Bio-X Institutes, Shanghai Jiao Tong University, Shanghai, China; ^2^Shanghai Key Laboratory of Psychotic Disorders, Brain Science and Technology Research Center, Shanghai Jiao Tong University, Shanghai, China; ^3^Shanghai Mental Health Center, Shanghai Jiao Tong University School of Medicine, Shanghai, China

**Keywords:** microRNA, biomarkers, 3D genome, Alzheimer's disease, machine learning

## Abstract

**Introduction:**

Alzheimer's disease (AD) is a type of neurodegenerative disease that has no effective treatment in its late stage, making the early prediction of AD critical. There have been an increase in the number of studies indicating that miRNAs play an important role in neurodegenerative diseases including Alzheimer's disease via epigenetic modifications including DNA methylation. Therefore, miRNAs may serve as excellent biomarkers in early AD prediction.

**Methods:**

Considering that the non-coding RNAs' activity may be linked to their corresponding DNA loci in the 3D genome, we collected the existing AD-related miRNAs combined with 3D genomic data in this study. We investigated three machine learning models in this work under leave-one-out cross-validation (LOOCV): support vector classification (SVC), support vector regression (SVR), and knearest neighbors (KNNs).

**Results:**

The prediction results of different models demonstrated the effectiveness of incorporating 3D genome information into the AD prediction models.

**Discussion:**

With the assistance of the 3D genome, we were able to train more accurate models by selecting fewer but more discriminatory miRNAs, as witnessed by several ML models. These interesting findings indicate that the 3D genome has great potential to play an important role in future AD research.

## 1. Introduction

### 1.1. Alzheimer's disease

Alzheimer's disease, one of the most common causes of dementia, is a progressive, persistent, and irreversible neurodegenerative disease affecting the normal functioning of the cerebral cortex and hippocampus (1). The causes of AD may consist of both genetic and epigenetic (environmentally acquired) factors. Risk gene germline mutations can only explain the pathogenesis of a small proportion of patients with AD. There are several hypotheses regarding the pathogenesis of AD, the more recognized ones being the Aβ amyloid (2), the Tau protein (3), the cholinergic depletion (4), the inflammaging (5), the oxidative stress (6), the mitochondrial damage (7), and the disrupted glycolipid metabolism (8). In addition, there are also some causal associations between these hypotheses.

Since patients with early AD (preclinical stage) often do not demonstrate significant disease symptoms, some patients may undergo a preclinical stage of up to 25 years (9). Several studies have analyzed the preventive effects of early screening for AD through meta-analysis and have shown that early intervention in the preclinical stage of AD can effectively slow down cognitive decline in subjects (10–12). Early screening for AD, therefore, is important as it can provide more information on the treatment options for patients with AD and psychological support for both the patients and their family members.

Due to that, the pathogenesis of AD may involve multiple pathogenic pathways acting together. As mentioned in previous studies, traditional pathophysiological biomarkers, in general, are ineffective in terms of specificity and sensitivity when combined, leaving the clinical choice of early AD biomarkers lacking (13). In recent years, several studies have reported that microRNAs (miRNAs) are closely associated with AD, but advanced prediction models and novel biological insights that can benefit the prediction are still insufficient. Therefore, in this study, we focus on miRNA and aim to explore their clinical biomarker potential in early AD detection from the perspective of the 3D genome (14, 15).

### 1.2. MicroRNA and AD

MicroRNAs are small non-coding RNAs (sncRNAs) consisting of 19–23 nucleotides (nt), and they are essential epigenetic and post-transcriptional regulators that cooperate with messenger RNA (mRNA). miRNAs are highly mobile and permeable, ubiquitous in the human brain and central nervous system, and the smallest eukaryotic nucleic acid (16–18). miRNAs are translocated and released into extracellular fluids, such as plasma/serum, cerebrospinal fluid, saliva, urine, tears, semen, and ovarian follicular fluid, and such secretable hormone-like miRNAs are known as extracellular miRNAs or circulating miRNAs (19). Extracellular miRNAs can be delivered to target cells *via* extracellular fluid circulation and regulate the corresponding cellular activities (20, 21); moreover, extracellular miRNAs are highly stable and can avoid degradation under stressful conditions such as storage at room temperature for up to 24 h and multiple freeze–thaw cycles (22). These properties indicate the potential and capability of utilizing miRNAs as biomarkers. In fact, some applications of miRNAs have already been explored, such as using them as biomarkers in neurological diseases like Parkinson's disease, Huntington's disease, amyotrophic lateral sclerosis, bipolar disorder, and schizophrenia (23, 24).

Studies have successfully revealed significant correlations between miRNA dysregulation and AD, such as miR-9, miR-34a, miR-125b, miR-146a, and miR-155 (25). Some have analyzed the metabolic pathways of Aβ and tau proteins and identified exosome miRNAs closely related to AD, including miR-193b, miR-342-3p, and miR-451a (26). Moreover, by analyzing the oxidative stress (OS) pathogenesis, researchers have found multiple affected miRNAs, such as miR-200c, miR-26b, miR-107, and miR-210 (27). These findings suggest the key role of miRNAs in AD pathogenesis. How to systematically discover more AD-related miRNAs suitable for AD early screening becomes the next challenge.

### 1.3. miRNA and 3D genome

As early as the 19th century, several studies observed that chromosomes in the nucleus exhibit a chromatin form and were kept in distinct and relatively fixed regions during interphase, leading to the concept of the “Chromosome Territory (CT)”. In CT, chromosome positions are relatively constant and differentially distributed between cells in which homologous chromosomes tend to separate from each other. In fact, only until the recent development of the chromosome conformation capture (3C) method (28) and its high-throughput method Hi-C (high-throughput chromosome conformation capture) (29), the concept of the 3D genome was systematically introduced.

Our previous studies on disease typing prediction discovered that adding chromatin 3D genome information into deep neural network models could significantly improve prediction accuracy (30, 31). This phenomenon is due to the fact that the 3D genome positions of disease-related DNAs and RNAs in the nucleus play an important role (e.g., the radius distance from chromatin to the center of the nucleus), which also suggests that the broader role of the 3D chromatin conformation in cell function and the mechanistic linkage between them are worth further investigation.

Although extracellular miRNAs are free-floating, the foremost step for their function is the repressive effect in the transcriptional phase. It has been hypothesized that the out-of-nucleus translocation of miRNAs in neurons can occur through the co-delivery of the AGO proteins and target mRNAs containing localization signals (32). In principle, the proximity of miRNAs to target DNA in the chromatin 3D spatial conformation is more efficient in utilizing cellular energy for physiological functions. Therefore, we believe that the 3D genome will play a critical role in boosting such interactions.

To summarize, we analyzed a causal chain of the “chromatin 3D conformation-driven cellular functional block” phenomenon: DNA co-localization → RNA co-expression → protein–protein interaction. These co-expressed RNAs will preferentially aggregate in the nucleus and be transported from specific nuclear pores into the cytoplasm. This allows for the efficient enrichment of small molecules in the cytoplasmic space. Such an approach increases the frequency of miRNA contact with target genes and accomplishes the regulation of genes with lower energy consumption, which is very much in line with the evolutionary rules. We suggest that the topologically associating domain (TAD)-like nuclear regions in the cell influence the cellular state and drive certain cellular behaviors; such blocks, which have a three-dimensional conformation and jointly regulate certain cellular functions, are “functional blocks”. Specifically, we believe that when a “functional block” is abnormally activated/inhibited, it will change the cellular state. When the DNA, the starting “puzzle”, is mutated or transcriptionally repressed, RNA transcription in the same “functional block” will also be abnormal, leading to abnormal protein expression and causing cellular dysfunction. We hypothesize that miRNAs that play essential disease-mediation functions also have certain spatially distributed properties. In this study, therefore, we optimize the miRNA-based AD prediction model by incorporating 3D genome information and further explore and discuss the value of applying 3D genomic information in AD early screening.

## 2. Methods

### 2.1. Dataset

The GSE120584 dataset adopted in this study was downloaded from the public database Gene Expression Omnibus (GEO). RNA data were extracted from the serum tissues of 1,309 Japanese individuals, containing 1,021 patients with AD and 288 normal controls (NCs). In this dataset, each miRNA signal value was standardized with the ratio of the average signal of the three internal control miRNA signals. The sample labels are indicated by 0 or 1, with 1 indicating patients with AD and 0 indicating normal control.

### 2.2. miRNA profiling

To quantify miRNA expression, we downloaded the RAW files of GSE120584 and generated the RNA expression matrix from them. We then converted the miRNA naming format to the latest miRBase V22 version by the miEAA 2.0 platform (33) and subsequently performed manual curation and validation. We mapped all miRNA tags to the human reference genome GRCh 37/hg19 using the R package (*bioMart*) which belongs to different compartments in the 3D genome. Due to the specificity of the miRNA biogenesis pathway, precursor miRNAs do not show one-to-one correspondence with mature miRNAs; thus, these miRNAs cannot be annotated uniquely and were excluded to ensure the miRNA uniqueness. After the annotation, transcripts with expression scores < 5 in < 1,000 samples were removed. Annotated miRNA quantification data were then adopted for Spearman correlation coefficient calculations and 3D clustering analysis. In the end, we obtained 1,605 valid miRNAs and 214 miRNAs with 3D information.

### 2.3. Acquisition of 3D coordinates of miRNAs

We constructed 3D genome models using a molecular dynamic approach based on the hESC cell line Hi-C data (34, 35), which generated 300 feasible conformational structures and selected the best-matched model as the 3D genome model for subsequent analyses. Then, by using the transcriptional start site (TSS) position of the miRNAs as an index, we matched miRNAs to the 3D coordinates in the intranuclear space.

### 2.4. Prediction model and feature selection

**Figure 1:**
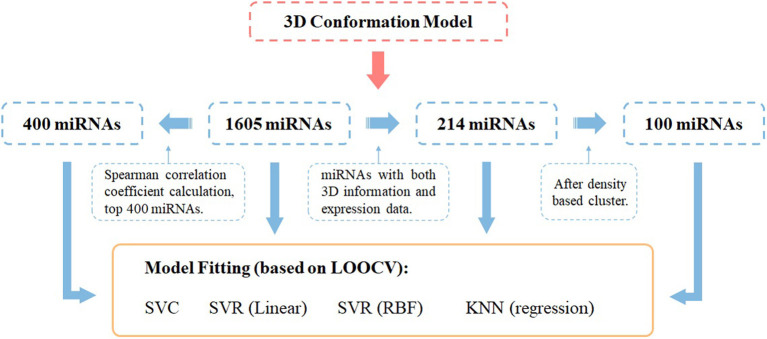
Workflow of miRNA panel selections and their AD score prediction effectiveness evaluated by different ML prediction models under the leave-one-out cross-validation framework.

Model construction was done by the python library *sklearn*, and DBSCAN was used for the density-based clustering method (eps = 3). All models were trained by leave-one-out cross-validation. The Spearman correlation coefficient is implemented with *scipy* in python. While evaluating the models, we set label = 0.5 as the cutoff, 0–0.5 is considered negative results, and 0.5–1 is considered positive results. The whole workflow of miRNA selection and model fitting has been shown in Figure 1.

## 3. Results

### 3.1. Clustering analysis of miRNAs on 3D positional

**Figure 2:**
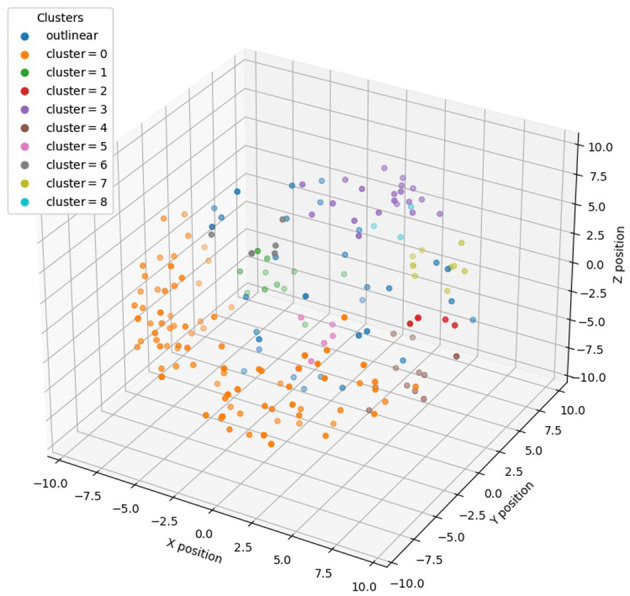
Distribution of different clusters of miRNAs in 3D genome space after DBSCAN clustering.

To subsequently combine the 3D distribution of miRNAs for feature selection, we performed density clustering DBSCAN of miRNAs with 3D information encoding, i.e., <*x, y, z*> coordinates, and obtained nine clusters with multiple miRNAs and 32 standalone clusters with single miRNAs. The clustering result is shown in Figure 2, and as it demonstrates, each miRNA cluster occupies a salient spatial territory.

### 3.2. Machine learning prediction model optimization

All samples were first divided into two groups according to disease type (0 or 1). We investigated three machine learning models under leave-one-out cross-validation (LOOCV): support vector classification (SVC), support vector regression (SVR), and k-nearest neighbors (KNNs). Both linear and RBF kernels were chosen for SVR.

First, we used all the annotated 1,605 miRNAs as features to perform predictions for AD; Table 1 demonstrates the effectiveness of each model using the all miRNAs feature panel. Subsequently, we performed a Spearman correlation coefficient (SCC)-based selection of the feature miRNAs and selected the top 400 miRNAs with the highest SCC score (*p* < 0.05). The results showed that prediction accuracies from all models increased to some extent (Table 1).

**Table 1:** Correlations between predicted AD values and true values under different models and different selected miRNA panels.

**miRNA**	**Whole genome miRNA expression (1,605)**						**Spearman correlation (top 400)**					
**Scores**	**Accuracy**	**Precision**	**Recall**	**F1_score**	**AUPR**	**AUC**	**Accuracy**	**Precision**	**Recall**	**F1_score**	**AUPR**	**AUC**
SVC	0.8342	0.8926	0.8952	0.8939	0.9087	0.8126	0.7785	0.8741	0.8364	0.8549	0.8837	0.7339
SVR (linear)	0.7217	0.8526	0.7668	0.8074	0.9054	0.7855	0.8171	0.842	0.9213	0.8799	0.9217	0.8253
SVR (rbf)	0.8851	0.879	0.9854	0.9292	0.9274	0.8514	0.8926	0.8862	0.9863	0.9335	0.9273	0.8564
KNN (regression)	0.8235	0.8297	0.9736	0.8959	0.9015	0.7703	0.8296	0.8342	0.9755	0.8993	0.9195	0.806632
**miRNA**	**3D and expression overlap (214)**						**3D cluster, DBSCAN (100)**					
**Scores**	**Accuracy**	**Precision**	**Recall**	**F1_score**	**AUPR**	**AUC**	**Accuracy**	**Precision**	**Recall**	**F1_score**	**AUPR**	**AUC**
SVC	0.8266	0.8825	0.8972	0.8898	0.9173	0.817	0.8449	0.8685	0.9442	0.9047	0.92	0.8186
SVR (linear)	0.8169	0.8169	0.9637	0.8843	0.9197	0.8215	0.7513	0.7465	0.9943	0.8528	0.9132	0.79
SVR (rbf)	0.8853	0.8797	0.9833	0.9286	0.9272	0.8571	0.8686	0.8692	0.9734	0.9183	0.9286	0.8553
KNN (regression)	0.8411	0.8362	0.9902	0.9067	0.9172	0.8002	0.8426	0.8376	0.9902	0.9075	0.9153	0.8052

To better investigate the ability of 3D genome information in contributing to the prediction models, we obtained the spatial coordinates of 214 miRNAs in the hESC nucleus by mapping the gene starting position on chromosomes. We proportionally selected the miRNAs with the highest Spearman correlation within each cluster as representatives of the clusters, yielding 100 miRNAs, and used them to train the model. Note that many informative miRNAs that could not be uniquely mapped to 3D genome space were discarded. The features obtained by such screening could still improve the prediction accuracies of the models, with the SVC model showing the most significant improvement.

**Figure 3:**
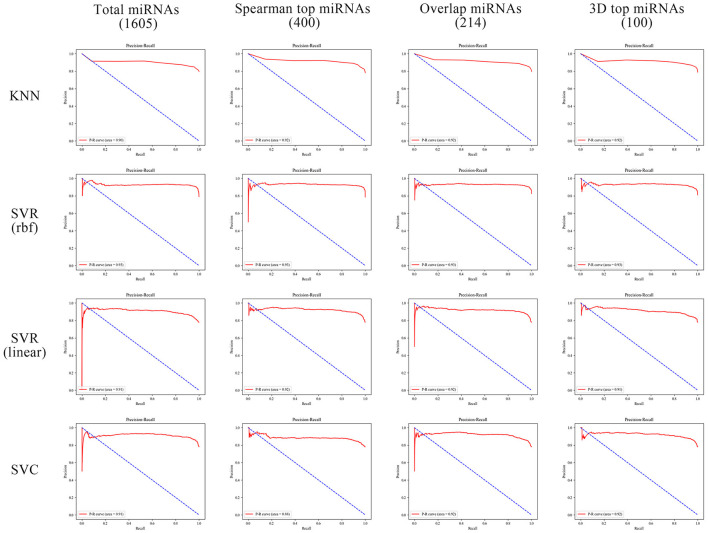
Precision–Recall curves of different ML models built using different miRNAs as prediction feature panels.

**Figure 4:**
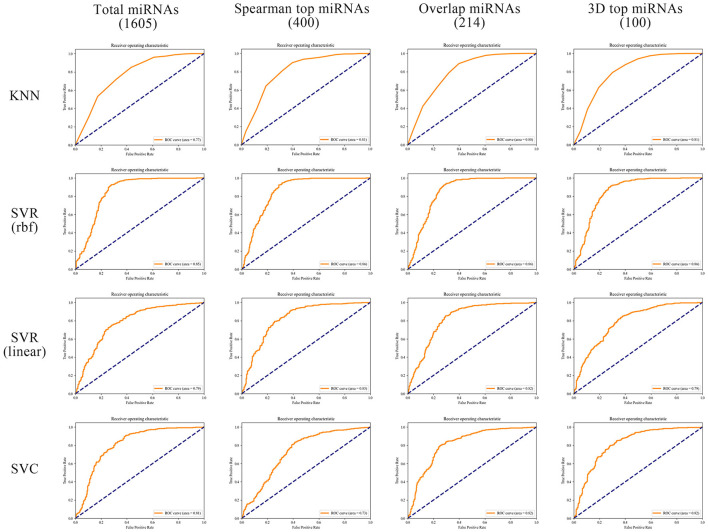
ROC curves of different ML models built using different miRNAs as prediction feature panels.

**Figure 5:**
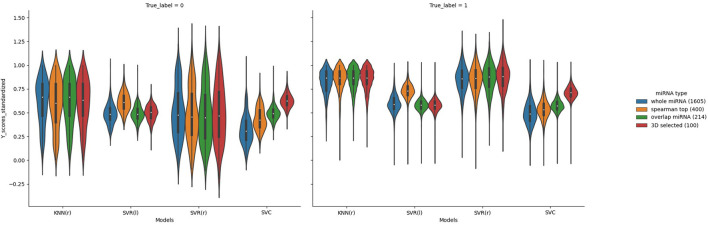
Violin plots of the predicted AD value distributions using different ML models.

In order to better compare the results under different features, we plotted the Precision–Recall curves (Figure 3) and ROC curves (Figure 4) for all models. We also plotted the violin plot (Figure 5) for the prediction results of different models, which demonstrated the effectiveness of incorporating 3D genome information into the AD prediction models.

### 3.3. Effects of epigenetic functions of miRNAs on AD

Indeed, the pathogenesis of AD is coherently linked to epigenetic phenomena, such as DNA methylation or non-coding RNA interference (36), while miRNAs play a role in the latter (37). During the feature selection, we further analyzed the miRNAs contributing to the prediction models. We found that miR-128 is one of the most contributing miRNAs; miR-128 was reported to downregulate PPAR-γ expression in mouse cortical neurons (MCNs) and Neuro2a (N2a) cells, which affects downstream NF-κB activity and, thus, triggers Aβ-mediated cytotoxicity (38). Similarly, we found another contributing miRNA miR-128, which was also reported to cause dysfunctional synaptic transmission between mossy cells (MCs) and somatostatin (SST) cells by inhibiting the normal function of the STIM2 gene (39). Furthermore, miR-210 in our predictor panel has even been found to play a role in the production of ROS in the brain with altered cholinergic neuronal states (40, 41). These findings provide essential reference information for the study of the pathogenesis of AD, and more relationships between differentially expressed miRNAs and corresponding targets in patients with AD are expected to be discovered in the future.

## 4. Conclusion and discussion

In this study, we analyzed the feature selection and machine learning prediction model optimization effects of using miRNA expression in AD prediction by incorporating 3D genome information. With the assistance of the 3D genome, we were able to train more accurate models by selecting fewer but more discriminatory miRNAs, as witnessed by all ML models, including SVC, SVR, and KNN. These interesting findings also indicate that the 3D genome has great potential to play an important role in future AD research.

MicroRNA is a typical epigenetic modulator undertaking multiple epigenetic mechanisms (42). Crosstalk between miRNAs and epigenetic regulation is important for neural development (37, 43). The enzymes of epigenetic modification processes can be regulated by miRNAs (44). Abnormal epigenetic regulation leads to abnormal miRNA expression, which further leads to the pathogenic mechanism of several malignancies (45). Specifically, manipulation of levels of mir-137, a miRNA associated with neuropsychiatric disorders in mice induces neurological abnormalities such as synaptic overgrowth, memory deficits, and repetitive behaviors (46).

Recent studies have further demonstrated that miRNAs can influence epigenetic phenomena by regulating the expression of DNA methylesterase. Several studies have found that the downregulation of miR-29 family (including miR-29a, miR-29b, and miR-29c) expression suppresses the expression of retinoblastoma-like protein 2 (Rbl2), which causes DNA methylation deficiency by inhibiting the activity of DNMT3a and DNMT3b (47, 48). It has also been shown that miR-17 and miR-20a in mammalian cells can induce heterochromatin formation in promoter regions with overlapping transcriptional functions and complementary to miRNA seed regions, further revealing a new mechanism of miRNA-regulated chromatin remodeling and gene transcription (49). These results suggest that there is a potentially complicated but strong connection between miRNA and 3D genome and worth in-depth exploration, and the analyses of neurodegenerative diseases such as AD from the novel perspective of 3D genome can be of great interest.

## Data availability statement

The original contributions presented in the study are included in the article/supplementary material, further inquiries can be directed to the corresponding authors.

## Ethics statement

Ethical review and approval was not required for the study on human participants in accordance with the local legislation and institutional requirements. Written informed consent from the patients/participants or patients/participants' legal guardian/next of kin was not required to participate in this study in accordance with the national legislation and the institutional requirements.

## Author contributions

KL, RC, and LL participated in the omics and computational experiments. MF and KS assisted computational experiments. KL provided the figures, tables, and drafted the manuscript. XL provided clinical suggestions about AD and biological insights into miRNA. YS and GH initiated this project, supervised the whole workflow, and edited and finalized the manuscript. All authors reviewed and proofread the manuscript and the experimental results.
